# Identification and Compensation Method of Unbalanced Error in Driving Chain for Rate-Integrating Hemispherical Resonator Gyro

**DOI:** 10.3390/s24134328

**Published:** 2024-07-03

**Authors:** Yiwei Sun, Zhennan Wei, Guoxing Yi, Ning Wang

**Affiliations:** Space Control and Inertial Technology Research Center, Harbin Institute of Technology, Harbin 150001, China; syw@stu.hit.edu.cn (Y.S.); ning.w@stu.hit.edu.cn (N.W.)

**Keywords:** rate-integrating hemispherical resonator gyro (RI-HRG), driving chain, unbalanced error, multi-loop control

## Abstract

The accuracy of the signal within a driving chain for the rate-integrating hemispherical resonator gyro (RI-HRG) plays a crucial role in the overall performance of the gyro. In this paper, a notable and effective method is proposed to realize the identification and compensation of the unbalanced error in the driving chain for the RI-HRG that improved the performance of the multi-loop control applied in the RI-HRG. Firstly, the assembly inclination and eccentricity error of the hemispherical resonator, the inconsistent metal conductive film layer resistance error of the resonator, the coupling error of the driving chain, and the parameter inconsistency error of the circuit components were considered, and the impact of these errors on the multi-loop control applied in the RI-HRG were analyzed. On this basis, the impact was further summarized as the unbalanced error in the driving chain, which included the unbalanced gain error, equivalent misalignment angle, and unbalanced equivalent misalignment angle error. Then, a model between the unbalanced error in the driving chain and a non-ideal precession angular rate was established, which was applicable to both single channel asynchronous control and dual channel synchronous control of the RI-HRG. Further, an unbalanced error identification and compensation method is proposed by utilizing the RI-HRG output with the virtual precession control. Finally, the effectiveness of the proposed method was verified through simulation and experiments in kind. After error compensation, the zero-bias instability of the RI-HRG was improved from 3.0950°/h to 0.0511°/h. The results of experiments in kind demonstrated that the proposed method can effectively suppress the non-ideal angular rate output caused by the unbalanced error in the driving chain for the RI-HRG, thereby further improving the overall performance of the RI-HRG.

## 1. Introduction

Hemispherical resonator gyros (HRGs) are simple and highly reliable angular motion sensors based on the Coriolis effect that belong to the category of Coriolis vibration gyroscopes (CVGs) [1,2]. The core sensing component of an HRG is a hemispherical resonator made of fused silica [3,4]. When the resonator is in a stable four-antinode vibration mode, the standing wave azimuth rotates with the angular rate input at a fixed precession factor [5]. On this basis, the carrier’s angular position and rate can be measured by monitoring the resonator’s four-antinode vibration mode. Owing to this, the core components of HRG typically consist of no more than three parts: the hemispherical resonator, the detection electrodes, and the driving electrodes, which further enhances its physical stability [6]. Furthermore, since the introduction of HRGs, they have been extensively used in aerospace applications, and their measurement accuracy and reliability have been fully validated in various deep-space exploration missions [7,8]. Initially, the detection and driving electrodes of HRGs were composed of two separate components [9], significantly increasing their manufacturing and assembly complexity and severely limiting their mass production. In recent years, the structure of HRGs have been further simplified by integrating the detection electrodes and driving electrodes, which has significantly increased their production and achieved their miniaturization, further reducing their volume, mass, and power consumption. These changes have expanded the potential applications of HRGs to numerous sectors, sparking a resurgence of interest in related technologies and further advancing their development [10].

With the development of HRGs and their related manufacturing technologies, the vibrating ring gyroscope (VRG) has emerged, which is also a type of micro-electro-mechanical system (MEMS) gyroscope. Initially, VRGs adopted a single ring structure: a single ring resonant structure connected to the anchor point through a supporting spring, where the electrodes can be freely arranged on the inner and outer sides of the ring [11]. However, multi ring structures are commonly used at present, which have higher symmetry compared to single ring structures and more flexible electrode arrangement schemes. Thus, the stray signals sensitive to environmental vibrations due to the asymmetric parameters of the resonant structures can be effectively suppressed and their static and dynamic performance can be further improved [12]. Moreover, a disk resonator gyroscope (DRG) can be thought of as an extension of a VRG. A DRG has multiple advantages, such as a moderate frequency, large effective mass, low anchor loss and so on, thus becoming the most attractive candidate for high-performance MEMS gyroscopes [13,14].

By comparison, HRGs have a longer history of development and the manufacturing accuracy of the core components can be better controlled, so that the performance of these high-precision products has been verified in deep space exploration missions. However, while the development time of VRGs is relatively short, VRGs are more conducive to batch processing and have great development potential. Furthermore, the main application areas of the two gyroscopes are currently different. HRGs are still mainly used in the aerospace and submarine fields, while VRGs are mainly used in emerging fields such as intelligent devices. Returning the focus to HRGs, they can be divided into force-to-rebalance hemispherical resonator gyros (FTR-HRGs) and rate-integrating hemispherical resonator gyros (RI-HRGs) according to their working modes. Generally, the standing wave azimuth of an FTR-HRG is controlled at a specific angle. Owing to this, the output range of the control signal limits the dynamic range of an FTR-HRG [15,16]. In contrast, an RI-HRG achieves stable standing wave control with a multi-loop control system that includes an amplitude control loop, an orthogonal control loop, and a frequency tracking loop, and the standing wave azimuth of the resonator is not constrained typically, so that it is also called a whole-angle hemispherical resonator gyro (WA-HRG) [17,18,19]. This endows RI-HRGs with a broader dynamic range, thereby rendering them a research hotspot in the field of inertia and gaining widespread application [10,20]. On this basis, the stability of the four-antinode vibration mode of the hemispherical resonator is critical to the measurement precision of RI-HRGs. Additionally, since the driving electrodes of RI-HRGs are circumferentially distributed [21], the control forces for each control loop must be reallocated to the driving electrodes based on the standing wave azimuth of the resonator. In this process, the detection accuracy of the standing wave azimuth and the consistency of the driving chain directly impact the control precision of the RI-HRG, further affecting the stability of the resonator vibration mode and the performance of the RI-HRG. Hence, the primary focus of current research on enhancing the accuracy of the multi-loop control is on the aforementioned two aspects.

By enhancing the detection accuracy of the standing wave azimuth, the control accuracy of an RI-HRG can be indirectly improved. In reference [22], Wang et al. analyzed the impact of detection errors in RI-HRGs caused by manufacturing errors and inconsistent circuit parameters in standing wave azimuth and gyroscope control systems. A detection error identification and compensation method based on nonlinear least squares was proposed, effectively improving the zero-bias instability and scale factor nonlinearity of HRGs. Sun et al. in reference [23] addressed the issue of additional angle drift caused by the detection symmetry errors in the whole-angle micro-shell resonator gyroscope. The sine and cosine components of the angle estimation errors were used to characterize the detection gain and angle error, respectively, with targeted compensation implemented, significantly enhancing the performance of the HRG. Reference [24] is based on the detection and driving principle of the equivalent capacitance in CVGs, studying the impact of nonlinearity on the standing wave azimuth of MEMS rate-integrating gyroscopes. A nonlinear correction for capacitive displacement detection was proposed by Hu et al., minimizing the impact of nonlinear errors and significantly reducing high-order drift errors in standing wave azimuths, further improving their detection accuracy. In reference [25], Asadian et al. derived critical design parameters of dual-shell micro-gyroscopes for survivability under harsh environments, which can also guide the design of HRGs.

Additionally, several scholars have conducted research on identifying and compensating for inconsistency errors in driving chains of HRGs. In reference [26], Vatanparvar et al. noticed that gain mismatch in actuation electronics causes interfere with the free precession of the resonator, causing additional angle measurement errors. On this basis, a method was proposed to distinguish the error due to mechanical asymmetry from the error caused by electronic device gain mismatch, individually identifying and compensating for the mismatches, which significantly enhanced the performance of the rate-integrating CVGs. However, the method only addressed the electronic-related gain mismatch in the driving chain of the gyro, leaving residual effects of inconsistency errors in the control accuracy caused by other factors. Chen et al. introduced a gain mismatch identification and compensation method using modal reversal for a 16-electrode whole-angle micro-hemispherical resonator gyroscope in reference [27]. The proposed method comprehensively realized the identification and compensation of gain mismatch for both the detection and driving chain, effectively mitigating the angular rate fluctuation error and angle bias error in the gyroscope. In Reference [28], Yan et al. considered the combined effects of detection, driving, system phase, and resonator mechanical errors on the performance of WA-HRGs. They proposed a self-excitation enabled decoupling, calibration, and compensation method for the above errors to enhance the overall performance of the gyro. However, the proposed method does not address the coupling issues between different driving chains, limiting the precision of error identification and compensation.

According to the existing research, the driving chain consistency of RI-HRG is crucial for the precision of multi-loop control, which also serves as the foundation for control optimization techniques and the implementation of angle dependent error compensation. Existing studies primarily address the inconsistency errors due to the imperfect assembly of hemispherical resonators, while research on identifying and compensating for unbalanced errors in the driving chain caused by circuit component parameter inconsistency error and signal coupling error remains insufficient, so that the identification of and compensation precision for unbalanced error in the driving chain for RI-HRG still needs to be enhanced.

This paper investigated the unbalanced error in driving chains within multi-loop controls applied to RI-HRGs and proposed an identification and compensation method for unbalanced error in the driving chain based on the virtual precession of a hemispherical resonator. Firstly, factors such as assembly inclination error, assembly eccentricity error, inconsistent metal conductive film layer resistance error, circuit coupling error, and inconsistent circuit component parameter error in the RI-HRG were taken into account. The influence of each factor on the driving chain was analyzed, and a model for unbalanced error in driving chains was established, encompassing an unbalanced gain error, equivalent misalignment angle, and unbalanced equivalent misalignment angle error. The error model proposed in the paper is applicable to RI-HRGs across various control strategies. Then, the identification and compensation method for the unbalanced error in the driving chain based on virtual precession control is proposed. With the virtual precession, the impact of the angle dependent error caused by non-ideal physical characteristics of the hemispherical resonator can be decoupled, further ensuring the precision of the identification and compensation method. Finally, the proposed method has been validated through experiments in kind. Due to the use of digital control circuits, the entire error identification and compensation process can be automatically implemented through specially designed software, further improving the efficiency and the accuracy of error identification and compensation. After error compensation, zero-bias instability, the key performance indicator of an RI-HRG, was improved from 3.0950°/h to 0.0511°/h, which confirmed the effectiveness of the proposed method. Meanwhile, the method is also applicable to CVGs operating in rate-integrating mode.

## 2. The Control System of RI-HRGs

The multi-loop control of an RI-HRG maintains the stable four-antinode vibration mode of a hemispherical resonator. The existing control schemes, while generally similar, comprising an amplitude control loop, orthogonal control loop, and frequency tracking loop, differ in their specific implementations. To meet subsequent error compensation needs, it may be necessary to incorporate additional control force outputs like virtual precession control. Furthermore, most HRGs adopt an eight-electrode structure that can be categorized into single-channel and dual-channel control schemes depending on the functionality of each electrode. To more effectively assess the impact of an unbalanced error in the driving chain on the control accuracy of RI-HRG, a brief overview of the multi-loop control is provided first.

### 2.1. The Multi-Loop Control of RI-HRG

**Figure 1:**
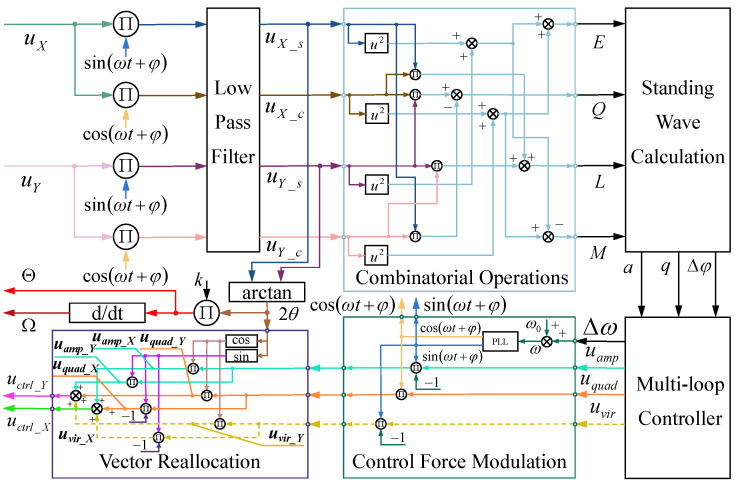
The multi-loop control of RI-HRG.

**Figure 2:**
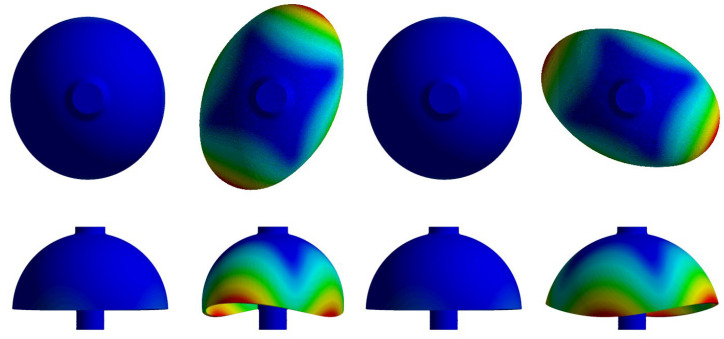
Finite element analysis of a hemispherical resonator.

An RI-HRG primarily comprises an amplitude control loop, a quadrature control loop, and a frequency tracking loop. The frequency tracking loop enables tracking of the intrinsic vibration frequency of the hemispherical resonator, generating the demodulation signal needed for the detection chain and the modulation signal needed for the driving chain. The amplitude and quadrature control loops are used to maintain the stable state of the standing wave within the resonator, establishing a foundation for precise angular position and rate outputs. Additionally, owing to the structure of the discrete electrodes, the control quantities for the amplitude and quadrature control loops must be reallocated based on the standing wave azimuth. Meanwhile, the control quantity used for the virtual precession also needs to be reallocated to the electrodes. The multi-loop control scheme applied in RI-HRG is illustrated in Figure 1:

By designing the detailed structure of the hemispherical resonator to separate the modal of the spherical shell and the rob of the resonator, the ideal resonator only operates in the four-antinode vibration mode of a hemispherical shell, as shown in Figure 2. Different colors within the model of the resonator represent different magnitude of the stress, which can also equivalent to the magnitude of the deformation, and the red part represents the maximum stress.

However, in the actual manufacturing process, it is difficult to ensure the complete consistency of the circumferential parameters of a hemispherical resonator, resulting in the hemispherical resonator only working approximately in the four-antinode vibration mode. In reality, it is a non-ideal traveling wave that can be equivalent to the main and auxiliary standing waves caused by the frequency split of the resonator. The phase and direction difference between the auxiliary standing wave and the main standing wave are all 90°, so it is also called the orthogonal wave. With a closed-loop control, the better the suppression effect of the amplitude of the orthogonal wave, the closer the hemispherical resonator is to the four-antinode vibration mode. The detection signals from the two sets of electrodes that are positioned 45° apart are 90° out of phase with each other ideally. Owing to this, the two sets of detection electrodes are designated as Channel X and Channel Y, and the detection signals $u_{X}$ and $u_{Y}$ are as follows:

(1) $$\left\{\begin{array}{c} u_{X}=a\cos2\theta \cos\left(\omega_{0}t+\varphi_{0}\right)-q\sin2\theta \sin\left(\omega_{0}t+\varphi_{0}\right)\\ u_{Y}=a\sin2\theta \cos\left(\omega_{0}t+\varphi_{0}\right)+q\cos2\theta \sin\left(\omega_{0}t+\varphi_{0}\right) \end{array}\right.$$

where a is the amplitude of the main standing wave within the hemispherical resonator, q is the amplitude of the orthogonal wave, $\omega_{0}$ is the intrinsic vibration frequency of the resonator, $\varphi_{0}$ is the initial phase of the vibration, and θ is the standing wave azimuth of the resonator. Further demodulating the detection signal yields the following parameters:

(2) $$\left\{\begin{array}{l} E=u_{X\_c}^{2}+u_{X\_s}^{2}+u_{Y\_c}^{2}+u_{Y\_s}^{2}=a^{2}+q^{2}\\ Q=2\left(u_{X\_c}\cdot u_{Y\_s}-u_{Y\_c}\cdot u_{X\_s}\right)=2aq\\ L=2\left(u_{X\_c}\cdot u_{X\_s}+u_{Y\_c}\cdot u_{Y\_s}\right)=\left(a^{2}-q^{2}\right)\sin2\Delta \varphi \\ M=u_{X\_c}^{2}-u_{X\_s}^{2}+u_{Y\_c}^{2}-u_{Y\_s}^{2}=\left(a^{2}-q^{2}\right)\cos2\Delta \varphi \end{array}\right.$$

where E and Q represent the demodulation quantities that encompass the amplitude of the main standing wave and the orthogonal wave, respectively; L and M represent the demodulation quantities that mainly encompass the phase of the demodulation reference signal; $u_{X\_c}$ and $u_{Y\_c}$ are derived by multiplying the detection signals from Channel X and Channel Y, respectively, with the cosine reference signal; $u_{X\_s}$ and $u_{Y\_s}$ are derived by multiplying the detection signals from Channel X and Channel Y, respectively, with the sine reference signal; and Δφ is the phase difference between the detection reference signal and the initial phase of the vibration. On this basis, the inputs for each controller in the multi-loop control of an RI-HRG can be articulated as:

(3) $$\left\{\begin{array}{l} q=\frac{1}{2}\left(\sqrt{E+Q}-\sqrt{E-Q}\right)\\ a=\sqrt{E-q^{2}}\\ \Delta \varphi =\frac{1}{2}\arctan\frac{L}{M} \end{array}\right.$$

In the multi-loop control of an RI-HRG, maintaining amplitude stability of the main standing wave while suppressing the amplitude of orthogonal wave is essential. During the operation of an RI-HRG, the main standing wave amplitude and orthogonal wave amplitude of the hemispherical resonator obtained from signal demodulation are used as inputs for the amplitude control loop and orthogonal control loop, respectively. The target value of the amplitude control loop is set with the consideration of factors like the resonator assembly space and the circuit gain, while the target value of the orthogonal control loop is set at zero. Additionally, the target value for the frequency tracking loop is determined by the specific definition of the modulation and demodulation reference signals in the control scheme, typically at 0° or 90°. Moreover, the prevalent virtual precession control application is an open-loop control. Then, the control signals will be modulated with the sine reference signal $u_{s}$ and cosine reference signal $u_{c}$ of the driving chain and further reallocated to the driving electrodes of Channel X and Channel Y based on the standing wave azimuth of the resonator. The control forces applied to the driving electrodes are as follows:

(4) $$\left\{\begin{array}{c} u_{amp\_X}=-u_{amp}u_{s}\cos2\theta \\ u_{amp\_Y}=-u_{amp}u_{s}\sin2\theta \\ u_{quad\_X}=-u_{quad}u_{c}\sin2\theta \\ u_{quad\_Y}=u_{quad}u_{c}\cos2\theta \\ u_{vir\_X}=u_{vir}u_{s}\sin2\theta \\ u_{vir\_Y}=-u_{vir}u_{s}\cos2\theta \end{array}\right.$$

where $u_{amp}$, $u_{quad}$ and $u_{vir}$ are control signals by the amplitude control loop, orthogonal control loop and virtual precession control, respectively; $u_{amp\_X}$, $u_{quad\_X}$ and $u_{vir\_X}$ are control forces applied to the driving electrodes of Channel X; and $u_{amp\_Y}$, $u_{quad\_Y}$ and $u_{vir\_Y}$ are control forces applied to the driving electrodes of Channel Y. Furthermore, the resultant forces $u_{ctrl\_X}$ and $u_{ctrl\_Y}$ that are applied to the driving electrodes of Channel X and Channel Y, respectively, can be expressed as:

(5) $$\left\{\begin{array}{c} u_{ctrl\_X}=u_{amp\_X}+u_{quad\_X}+u_{vir\_X}=-u_{amp}u_{s}\cos2\theta -u_{quad}u_{c}\sin2\theta +u_{vir}u_{s}\sin2\theta \\ u_{ctrl\_Y}=u_{amp\_Y}+u_{quad\_Y}+u_{vir\_Y}=-u_{amp}u_{s}\sin2\theta +u_{quad}u_{c}\cos2\theta -u_{vir}u_{s}\cos2\theta \end{array}\right.$$

As described above, the multi-loop control of an RI-HRG has been realized. Moreover, based on the specific functionality of each electrode, the control schemes can be further categorized into single-channel and dual-channel schemes.

### 2.2. The Single-Channel Control of RI-HRG

**Figure 3:**
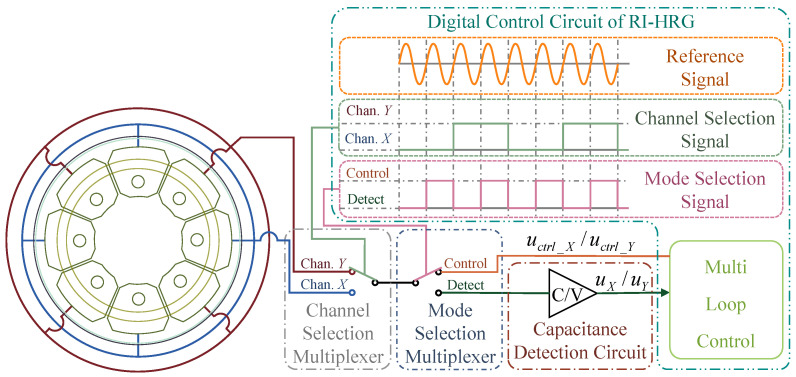
Single-channel control of RI-HRG.

A single-channel control scheme of an RI-HRG is a widely adopted control scheme. It utilizes several multiplexers to realize time-division multiplexing within a single detection and driving chain, sequentially fulfilling the signal detection and driving in Channel X and Channel Y according to a specific time sequence [29]. Consequently, the single-channel scheme effectively mitigates unbalanced errors in the detection and driving chains between Channel X and Channel Y caused by circuit disparities. Moreover, it enhances the utilization rate of each electrode during the detection and driving process, which can also partly suppress the impact of manufacturing defects within the hemispherical resonator. However, the use of multiplexers necessitates consideration of signal settling times in the digital control circuit, which constrains the frequency of the control system and causes additional signal delay errors, leading to non-ideal standing wave drift. Additionally, the use of multiplexers adversely affects the circuit’s overall stability and signal-to-noise ratio. A schematic diagram of a single-channel control scheme is displayed in Figure 3.

In Figure 3, the timing control logic of the multiplexers based on the reference signal generated by the frequency tracking loop is illustrated, and RI-HRG will sequentially realize the detection of Channel X, the driving of Channel X, the detection of Channel Y and the driving of Channel Y. It should be noted that Figure 3 primarily illustrates the timing control logic of the multiplexers, thereby simplifying the processing of the signals for the adjacent 90° electrodes in both the detection and driving chain. Ideally, the detection signals obtained by two electrodes 90° apart have the same amplitude but opposite signs. Likewise, the driving signals applied to electrodes 90° apart should also be differential signals. Therefore, simple processing is needed in the circuit implementation.

### 2.3. The Dual-Channel Control of RI-HRG

**Figure 4:**
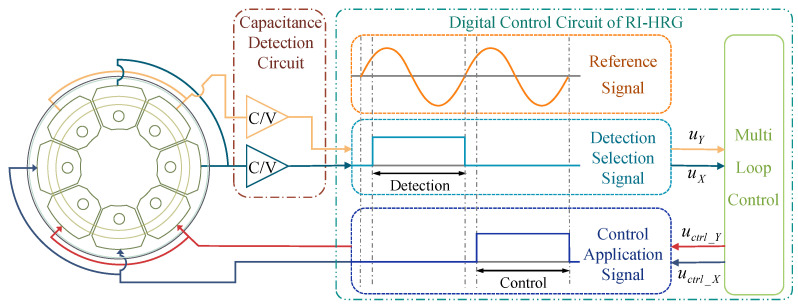
The dual-channel control of RI-HRG.

The dual-channel control scheme is developed from the control system of three-piece HRGs. Each electrode serves a specific function and is free from additional functions, so that Channel X and Channel Y can active simultaneously [30,31,32]. The dual-channel control of RI-HRG is shown in Figure 4.

As shown in Figure 4, the detection and driving processes are separated from the digital control circuit to further enhance the accuracy of the detection and driving of RI-HRG. The dual-channel control has a simpler circuit, which also enhances the reliability of the RI-HRG. However, the coupling effect of the driving signals in driving the chains cannot be avoided. Indeed, the function of each electrode should be decided after comprehensive consideration. The use of electrodes 180° apart helps to homogenize the impact of assembly errors, while using electrodes 90° apart can effectively reduce common mode interference within the circuits. Owing to this, the application scheme of the electrodes may vary with the specific control scheme.

## 3. The Unbalanced Error in Driving Chains for RI-HRG

An RI-HRG can be divided into two main parts: the sensing head and the mixed analog–digital control circuit. The sensing head primarily consists of a hemispherical resonator and a flat electrode, both made of fused silica. With the continuous improvement of manufacturing processes, the performance of the resonator has been significantly enhanced, laying the foundation for RI-HRG performance improvement. However, during the manufacturing process, assembly errors that can affect the detection and driving precision still occur. Besides, the detection and driving precision can also be influenced as it may be difficult to ensure complete consistency of the circuit parameters. To effectively enhance the control precision of the RI-HRG, the unbalanced errors in the driving chain caused by both the sensing head and the mixed analog–digital control circuit were analyzed, laying the groundwork for future error identification and compensation.

### 3.1. The Unbalanced Error in Driving Chain Caused by the Sensing Head of RI-HRG

**Figure 5:**
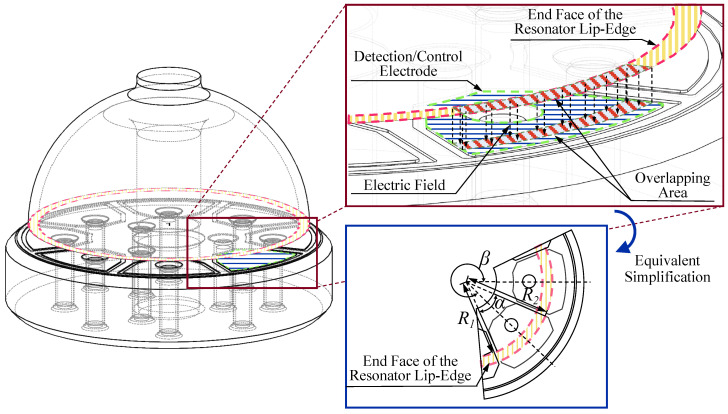
The equivalent flat capacitor of RI-HRG.

The detection and driving processes of RI-HRG are based on the equivalent flat capacitor, which is formed between the end face of the resonator lip-edge and the flat electrode, as shown in Figure 5. In Figure 5, $R_{1}$ represents the inner spherical radius of the resonator, $R_{2}$ represents the outer spherical radius, α denotes the electrode span angle, and β indicates the electrode center angle.

As the hemispherical resonator is in the four-antinode vibration mode, the distance between the end face of the resonator lip-edge and the flat electrode is periodically changed. Ideally, if d represents the distance between the end face of the resonator lip-edge and the flat electrode, then the capacitance of the equivalent flat capacitor can be calculated as:

(6) $$C=\frac{\alpha \left(R_{2}^{2}-R_{1}^{2}\right)}{8\pi k\left[d-\left(x\cos2\beta +y\sin2\beta \right)\right]},\beta =0,\frac{\pi }{4},\cdots ,\frac{7\pi }{4}$$

where, k is the Coulomb constant, $k=8.9876\times 10^{9}N\cdot m/C$.

**Figure 6:**
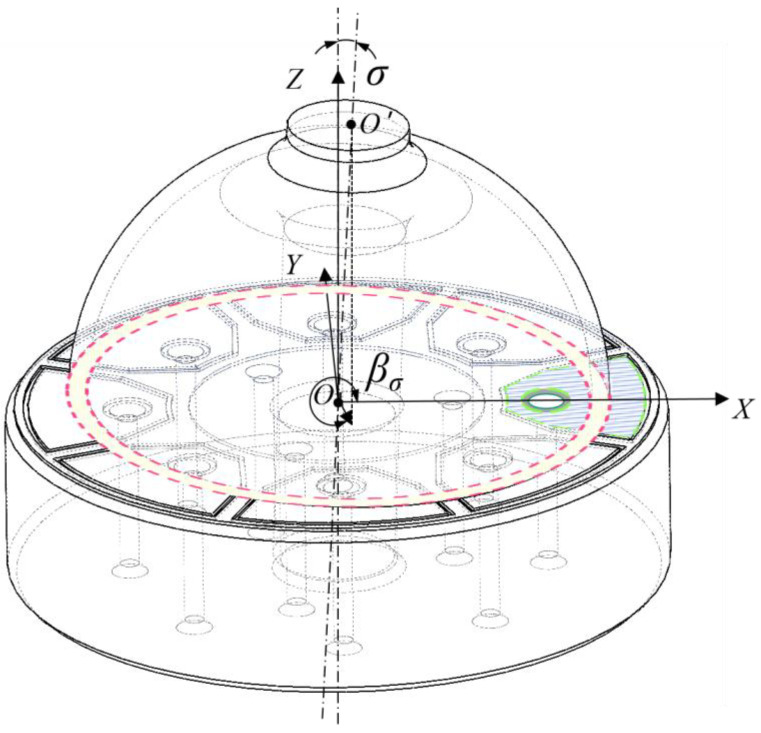
The assembly inclination error of RI-HRG.

However, due to the constraints of the manufacturing process, the hemispherical resonator may not be perpendicular to the flat electrode. Consequently, the distance between the end face of the resonator lip-edge and the flat electrode is no longer consistent but is influenced by the inclination error σ and the inclination azimuth error $\beta_{\sigma }$ as shown in Figure 6, resulting from the imperfect assembly of the hemispherical resonator.

The capacitance with assembly inclination error can be expressed as:

(7) $$C=\frac{\alpha \left(R_{2}^{2}-R_{1}^{2}\right)}{8\pi k\left[d-\sigma R\cos\left(\beta -\beta_{\sigma }\right)-\left(x\cos2\beta +y\sin2\beta \right)\right]},\beta =0,\frac{\pi }{4},\cdots ,\frac{7\pi }{4}$$

Assuming that the control voltage applied to the electrode is $u_{f}$, then the control force applied to the hemispherical resonator is:

(8) $$F_{\beta }=-\frac{\partial }{\partial d}\left(\frac{1}{2}Cu_{f}^{2}\right)\approx G_{\beta }u_{f}^{2}$$

where $G_{\beta }$ is the voltage-to-driving-force gain with the consideration of the assembly inclination error, and its form is as follows:

(9) $$G_{\beta }=-\frac{\alpha \left(R_{2}^{2}-R_{1}^{2}\right)}{16\pi k{\left[d-\sigma R\cos\left(\beta -\beta_{\sigma }\right)\right]}^{2}}-\frac{\alpha \left(R_{2}^{2}-R_{1}^{2}\right)\left(x\cos2\beta +y\sin2\beta \right)}{8\pi k{\left[d-\sigma R\cos\left(\beta -\beta_{\sigma }\right)\right]}^{3}}$$

In practical multi-loop control of RI-HRGs, the control voltage $u_{f}$ essentially represents the voltage difference between the end face of the resonator lip-edge and the flat electrode, which is composed of two components, the DC high voltage $u_{DC}$ that is applied to the end face of the resonator lip-edge and the control signals $u_{ctrl\_X}$ and $u_{ctrl\_Y}$ that are applied to the driving electrodes in Channel X and Channel Y, respectively. The control signals are modulated by the sine and cosine reference signals generated by the frequency tracking loop. Consequently, they can be considered as AC signals with the same frequency as the vibration of the resonator. The control voltage in Channel X and Channel Y, $u_{f\_X}$ and $u_{f\_Y}$, respectively, are as follows:

(10) $$\left\{\begin{array}{c} u_{f\_X}=u_{DC}+u_{ctrl\_X}\\ u_{f\_Y}=u_{DC}+u_{ctrl\_Y} \end{array}\right.$$

Due to variations in the specific control scheme, the electrodes used in the driving chain may also change. However, the voltage-to-driving-force gain for each electrode can be directly calculated by substituting the electrode center angle into Equation (9). Assuming that the voltage-to-driving-force gain in Channel X and Channel Y are $G_{\beta \_X}$ and $G_{\beta \_Y}$, respectively, then the driving forces generated by the driving electrodes in Channel X and Channel Y, $F_{X}$ and $F_{Y}$, can be further represented as:

(11) $$\left\{\begin{array}{c} F_{X}=G_{\beta \_X}\left(u_{DC}^{2}+\frac{1}{2}u_{ctrl\_X}^{2}+2u_{DC}u_{ctrl\_X}\right)\\ F_{Y}=G_{\beta \_Y}\left(u_{DC}^{2}+\frac{1}{2}u_{ctrl\_Y}^{2}+2u_{DC}u_{ctrl\_Y}\right) \end{array}\right.$$

In Equation (11), the DC high voltage and the square term of the AC control signal constitute the DC and high-frequency components of the control force. In multi-loop control of the RI-HRG, the DC component in the control force has an impact on the stiffness of the resonator; however, the impact will be significantly weakened or even eliminated by the orthogonal control. In addition, the high-frequency component in the control force does not have an effective driving effect on the resonator. Thus, only the control force with the same frequency as the resonator, the product term of the DC high voltage and the AC control signal, is applicable in multi-loop control of the RI-HRG. On this basis, the control force can be further simplified as follows:

(12) $$\left\{\begin{array}{c} F_{X}=2G_{\beta \_X}u_{DC}u_{ctrl\_X}\\ F_{Y}=2G_{\beta \_Y}u_{DC}u_{ctrl\_Y} \end{array}\right.$$

As shown in Equation (12), the assembly inclination of the resonator leads to a deviation in the actual driving force generated by the driving electrodes. Assuming that the control force gain in Channel X is $G_{\sigma }$, and the relative error of the control force gain in Channel Y is $\Delta G_{\sigma }$, then the control forces are as follows:

(13) $$\left[\begin{array}{c} F_{X}\\ F_{Y} \end{array}\right]=\left[\begin{array}{cc} G_{\sigma } & 0\\ 0 & G_{\sigma }\left(1+\Delta G_{\sigma }\right) \end{array}\right]\left[\begin{array}{c} u_{DC}u_{ctrl\_X}\\ u_{DC}u_{ctrl\_Y} \end{array}\right]$$

**Figure 7:**
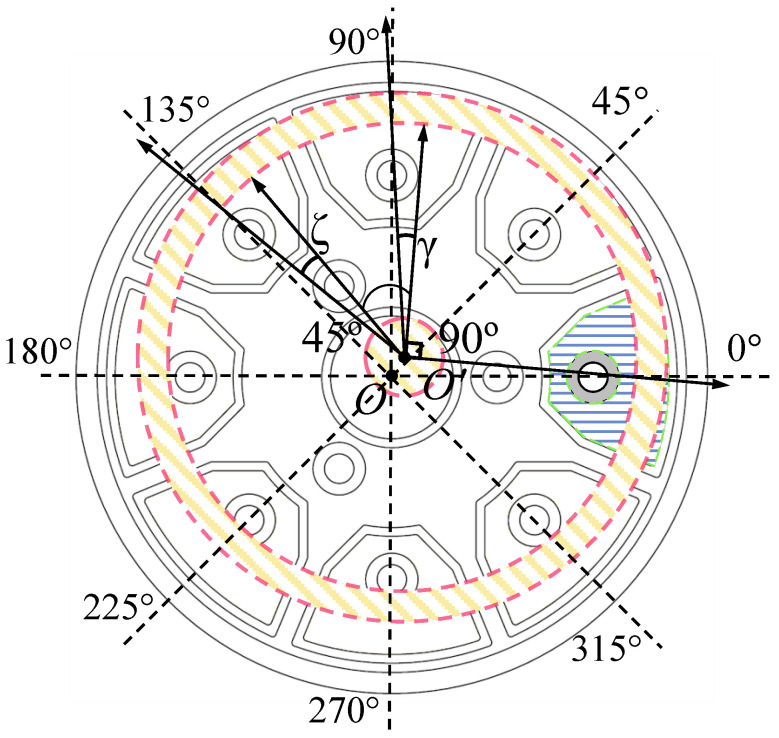
The assembly eccentricity error of RI-HRG.

In the assembly process of the hemispherical resonator and the flat electrode, there will also be an assembly eccentricity error besides the assembly inclination error. Due to the assembly eccentricity error, the sensitive axis of the hemispherical resonator does not align with the center of the flat electrode, as shown in Figure 7.

With the impact of the assembly eccentricity error, the center angles of the electrodes used in the driving chain shift from $90^{\circ }$ and $135^{\circ }$ to $90^{\circ }+\gamma$ and $135^{\circ }+\zeta$, respectively. In fact, the diameter of the assembly hole in the center of the flat electrode is only marginally larger than that of the support rod of the hemispherical resonator, so that an adequate space is kept to fill with adhesive while assembling. Although it may cause assembly eccentricity error, the resultant electrode deviation angle is a small quantity. Therefore, the deviation angle between different channels can be approximately considered consistent. If the electrode deviation angle caused by the assembly eccentricity error is λ, then the control forces will change to the following form under the influence of the assembly eccentricity error:

(14) $$\left[\begin{array}{c} F_{X}\\ F_{Y} \end{array}\right]=\left[\begin{array}{cc} \cos2\lambda & -\sin2\lambda \\ \sin2\lambda & \cos2\lambda \end{array}\right]\left[\begin{array}{cc} G_{\sigma } & 0\\ 0 & G_{\sigma }\left(1+\Delta G_{\sigma }\right) \end{array}\right]\left[\begin{array}{c} u_{DC}u_{ctrl\_X}\\ u_{DC}u_{ctrl\_Y} \end{array}\right]$$

**Figure 8:**
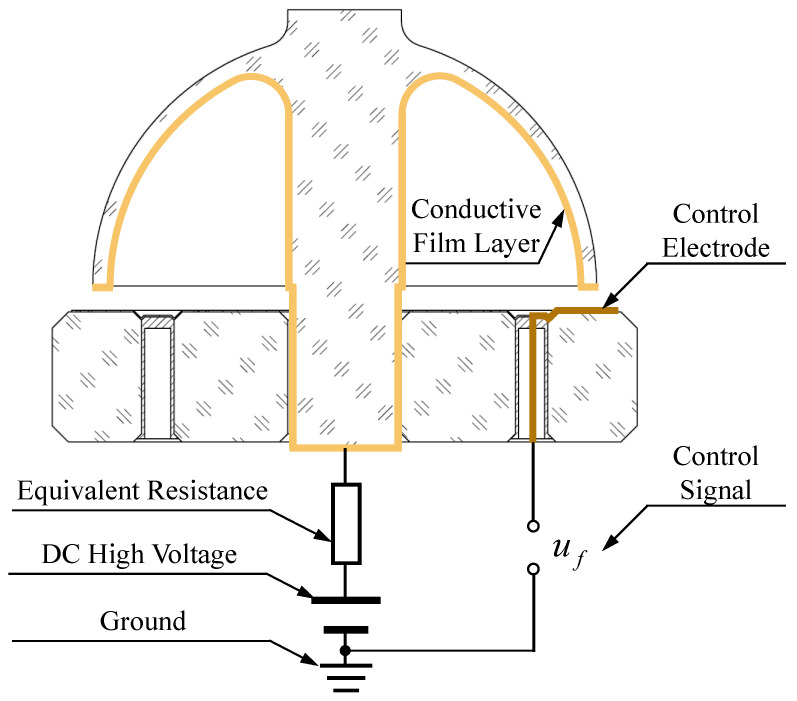
The equivalent driving chain in the sensing head of RI-HRG.

In addition to the driving errors caused by the assembly process of the RI-HRG, the fabrication of the metal conductive film layer on the hemispherical resonator also has an impact on the driving of the RI-HRG. The metal conductive film layer is coated on the inner surface and support rod of the resonator, and during this process, the influence of the roughness of the inner surface on the continuity of the metal conductive film layer and the influence of the thickness of the metal film layer on the quality factor of the resonator ought to be taken into consideration. Owing to this, the uniformity of the metal conductive film layer is not ideal due to the influence of the inner surface roughness of the resonator and the magnetron sputtering coating process, which is reflected in the varying resistance between the end face of the support rod and the points along the end face of the resonator lip-edge. The driving chain in the sensing head of RI-HRG is shown in Figure 8.

The equivalent resistance shown in Figure 8 represents the resistance between the end face of the resonator lip-edge and the end face of the support rod. Each point corresponds to a unique equivalent resistance, resulting in varying DC high voltages along the end face of the resonator lip-edge projected to the driving electrodes in Channel X and Channel Y. Ideally, ignoring the equivalent resistance, if the input DC high voltage at the support rod is $u_{DC}$, then the DC high voltage at the end face of the resonator lip-edge is also $u_{DC}$. However, due to the imperfect manufacturing of the film layer, the actual high voltage at the end face of the resonator lip-edge corresponding to the driving electrodes in Channel X and Channel Y will be reduced to $u_{DC\_X}$ and $u_{DC\_Y}$, respectively. Under this circumstance, the control force can be expressed as follows:

(15) $$\left[\begin{array}{c} F_{X}\\ F_{Y} \end{array}\right]=\left[\begin{array}{cc} \cos2\lambda & -\sin2\lambda \\ \sin2\lambda & \cos2\lambda \end{array}\right]\left[\begin{array}{cc} G_{\sigma } & 0\\ 0 & G_{\sigma }\left(1+\Delta G_{\sigma }\right) \end{array}\right]\left[\begin{array}{c} u_{DC\_X}u_{ctrl\_X}\\ u_{DC\_Y}u_{ctrl\_Y} \end{array}\right]$$

Furthermore, it can be seen from Equation (15) that the influence of the film layer within the resonator is similar to the influence of the assembly inclination error. Taking the DC high voltage in Channel X, $u_{DC\_X}$, as the reference, and using $G_{R}$ to represent it, further assuming that the DC high-voltage error in Channel Y relative to Channel X is $\Delta G_{R}$, then the control forces can be expressed as follows:

(16) $$\left[\begin{array}{c} F_{X}\\ F_{Y} \end{array}\right]=\left[\begin{array}{cc} \cos2\lambda & -\sin2\lambda \\ \sin2\lambda & \cos2\lambda \end{array}\right]\left[\begin{array}{cc} G_{\sigma } & 0\\ 0 & G_{\sigma }\left(1+\Delta G_{\sigma }\right) \end{array}\right]\left[\begin{array}{cc} G_{R} & 0\\ 0 & G_{R}\left(1+\Delta G_{R}\right) \end{array}\right]\left[\begin{array}{c} u_{ctrl\_X}\\ u_{ctrl\_Y} \end{array}\right]$$

By re-organizing Equation (16), the model from the ideal control signals to the driving forces is obtained, which has considered the impact caused by the assembly inclination error, assembly eccentricity error, and inconsistent conductive film layer resistance error of the hemispherical resonator assembly, and the model is shown as follows:

(17) $$\left[\begin{array}{c} F_{X}\\ F_{Y} \end{array}\right]=\left[\begin{array}{cc} G_{\sigma }G_{R}\cos2\lambda & -G_{\sigma }G_{R}\left(1+\Delta G_{\sigma }\right)\left(1+\Delta G_{R}\right)\sin2\lambda \\ G_{\sigma }G_{R}\sin2\lambda & G_{\sigma }G_{R}\left(1+\Delta G_{\sigma }\right)\left(1+\Delta G_{R}\right)\cos2\lambda \end{array}\right]\left[\begin{array}{c} u_{ctrl\_X}\\ u_{ctrl\_Y} \end{array}\right]$$

The model of the unbalanced error in the driving chain caused by the sensing head of RI-HRG is shown in Equation (17), which is the main driving error in the single channel control scheme and is part of the driving error in the dual-channel control scheme, so that the identification and compensation for the unbalanced error in the driving chain caused by the sensing head is crucial for enhancing the performance of RI-HRG.

### 3.2. The Unbalanced Error in the Driving Chain Caused by the Control Circuit of RI-HRG

In the dual-channel control scheme of RI-HRG, the unbalanced error in driving chain is caused not only by the sensing head but also by the control circuit. In the dual-channel control scheme of RI-HRG, the driving signals in Channel X and Channel Y are simultaneously applied to the driving electrodes through independent signal chains, so that it is difficult to maintain complete consistency in the parameters of the circuit components used. In the driving chain, the output signal from the digital controller to the driving electrodes needs to go through digital to analog converters, operational amplifiers, resistors, and capacitors, and their inconsistency parameters will lead to inconsistent gain of the voltage at the driving electrodes relative to the digital output. At this point, assuming that the control circuit gain of the driving chain in Channel X is $G_{e}$, and setting it as the reference, further defining the control circuit gain error as $\Delta G_{e}$, on this basis, when the outputs of the digital controllers for Channel X and Channel Y are $u_{ctrl\_X\_O}$ and $u_{ctrl\_Y\_O}$, respectively, with the consideration of the circuit component parameter error, the control signals applied to the driving electrodes are as follows:

(18) $$\left[\begin{array}{c} u_{Ctrl\_X}\\ u_{Ctrl\_Y} \end{array}\right]=\left[\begin{array}{cc} G_{e} & 0\\ 0 & G_{e}\left(1+\Delta G_{e}\right) \end{array}\right]\left[\begin{array}{c} u_{Ctrl\_X\_O}\\ u_{Ctrl\_Y\_O} \end{array}\right]$$

**Figure 9:**
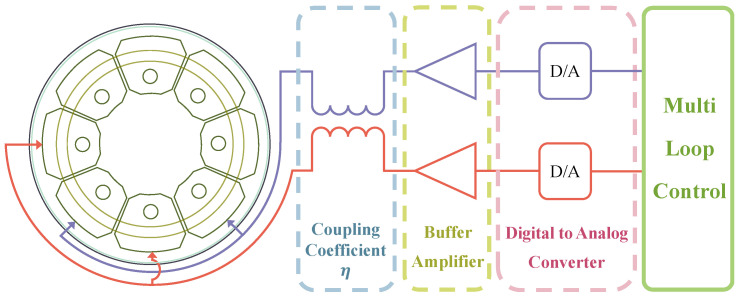
The coupling error in control circuit of RI-HRG.

Besides, in the dual-channel control scheme of RI-HRG, the driving signals in Channel X and Channel Y are applied to the driving electrodes simultaneously. However, as shown in Figure 9, completely shielding the signals within the driving chain is difficult, which results in signal coupling, further compromising the control precision.

As shown in Figure 9, assuming that the coupling coefficient of the signal in the driving chain is η, the control signals actually applied to the driving electrodes can be further expressed as follows:

(19) $$\left[\begin{array}{c} u_{Ctrl\_X}\\ u_{Ctrl\_Y} \end{array}\right]=\left[\begin{array}{cc} 1 & \eta \\ \eta & 1 \end{array}\right]\left[\begin{array}{cc} G_{e} & 0\\ 0 & G_{e}\left(1+\Delta G_{e}\right) \end{array}\right]\left[\begin{array}{c} u_{Ctrl\_X\_O}\\ u_{Ctrl\_Y\_O} \end{array}\right]=\left[\begin{array}{cc} G_{e} & \eta G_{e}\left(1+\Delta G_{e}\right)\\ \eta G_{e} & G_{e}\left(1+\Delta G_{e}\right) \end{array}\right]\left[\begin{array}{c} u_{Ctrl\_X\_O}\\ u_{Ctrl\_Y\_O} \end{array}\right]$$

The unbalanced error in the driving chain caused by the control circuit is the main driving error in the dual-channel control scheme. While component selection and circuit shielding can partly mitigate its impact, they also lead to higher manufacturing costs for RI-HRG, and furthermore, are not wholly effective. To further improve the control accuracy and overall performance of RI-HRG, the identification and compensation method needs to comprehensively consider the impact of the unbalanced error in the driving chain caused by the sensing head and the control circuit.

### 3.3. The Model of Unbalanced Error in Driving Chain

With the foundation of the existing research, both the single-channel control scheme and the dual-channel control scheme of RI-HRG offer distinct advantages. The aforementioned analysis reveals differing main components of driving errors in the two control schemes. On this basis, to enhance the efficacy and versatility of the identification and compensation method of the unbalanced error in the driving chain for the RI-HRG, a comprehensive consideration of the unbalanced error caused by the sensing head and the control circuit is given, and a unbalanced error model is established. As the errors identified in the analysis are generally small quantities, the high-order terms resulting from the multiplication can be disregarded, and then the simplified model of the unbalanced error in the driving chain is presented as follows:

(20) $$\left[\begin{array}{c} F_{X}\\ F_{Y} \end{array}\right]=G\left[\begin{array}{cc} 1 & \eta -\tan2\lambda \\ \eta +\tan2\lambda & 1+\Delta G \end{array}\right]\left[\begin{array}{c} u_{Ctrl\_X\_O}\\ u_{Ctrl\_Y\_O} \end{array}\right]$$

where, $G=G_{e}G_{\sigma }G_{R}\cos2\lambda$, is the reference gain of the control forces relative to the control outputs and $\Delta G=\Delta G_{e}+\Delta G_{\sigma }+\Delta G_{R}$ is the unbalanced gain error. With the impact of the assembly eccentricity error and the coupling error, the control forces applied to the driving electrodes in Channel X and Channel Y are no longer orthogonal in position, which is equivalent to introducing an angle error, so that the coupling coefficient η is replaced by tan2δ, which has the same range of values and is consistent with the form of the assembly eccentricity error and much easier to explain. On this basis, 2δ is further defined as the equivalent misalignment angle and 2λ is defined as the unbalanced equivalent misalignment angle error. Further, the Equation (20) can be rewritten as follows:

(21) $$\left[\begin{array}{c} F_{X}\\ F_{Y} \end{array}\right]=G\left[\begin{array}{cc} 1 & \tan2\delta -\tan2\lambda \\ \tan2\delta +\tan2\lambda & 1+\Delta G \end{array}\right]\left[\begin{array}{c} u_{Ctrl\_X\_O}\\ u_{Ctrl\_Y\_O} \end{array}\right]$$

Equation (21) presents the model of the unbalanced error in the driving chain with the comprehensive consideration of the influence from the manufacturing process and the control scheme within the RI-HRG. Moreover, the model is effective for RI-HRG across various electrode application schemes and control schemes.

In summary, the unbalanced error causes a deviation between the actual control forces and the expected control forces from the multi-loop control, resulting in a mismatch between the actual control forces in Channel X and Channel Y. This is mainly manifested in the unbalanced gain and the non-orthogonal direction of the applied control forces, which will have a negative impact on the vibration state of the hemispherical resonator.

Based on this model, the impact of non-ideal control forces on the standing wave of the hemispherical resonator was further analyzed, thus achieving the identification and compensation of the unbalanced error in the driving chain.

## 4. The Identification and Compensation Method

A conclusion can be obtained that although the causes of the unbalanced errors in the driving chain are different, since they are closely tied to the structure and control scheme of the RI-HRG, the impact on the actual driving forces applied to the driving electrodes are the same, as shown in Equation (21). On this basis, the impact of the unbalanced error on the standing wave is analyzed, and the output of the RI-HRG is used as the observation to identify the unbalanced error. The identification results are further applied in the digital control circuits to achieve compensation for the error. Equation (21) has considered most of the errors that affect the driving accuracy in the RI-HRG, which ensures the subsequent identification and compensation method with high applicability.

### 4.1. The Influencing Mechanism of the Unbalanced Error in the Driving Chain

**Figure 10:**
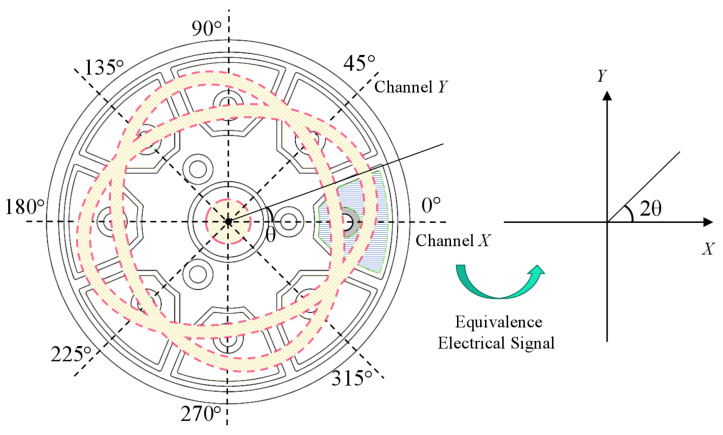
The correspondence between mechanical azimuth and electrical azimuth.

In RI-HRG, the standing wave azimuth of the hemispherical resonator is not controlled due to the four-antinode vibration mode. When the standing wave azimuth relative to the $0^{\circ }$ electrode is θ, the electrical azimuth directly obtained by the detection signal will be 2θ, as shown in Figure 10.

In multi-loop control of the RI-HRG, the amplitude control, the orthogonal control and the frequency tracking control are all closed-loop controls, so that the impact of the driving errors can be effectively suppressed. However, the precession control is an open-loop control, and its modulation scheme is the same as the amplitude control. Thus, the amplitude control will generate a non-ideal virtual precession control signal with the impact of the driving errors, which will directly affect the standing wave azimuth of the hemispherical resonator and further affect the output accuracy.

Ideally, as shown in Equation (4), the amplitude control signal obtained by vector reallocation of the RI-HRG can be expressed as follows:

(22) $$\left\{\begin{array}{c} u_{amp\_X}=-u_{amp}u_{s}\cos2\theta \\ u_{amp\_Y}=-u_{amp}u_{s}\sin2\theta \end{array}\right.$$

The amplitude control forces in Channel X and Channel Y with the impact of the unbalanced error in the driving chain can be expressed as follows:

(23) $$\left[\begin{array}{c} F_{amp\_X}\\ F_{amp\_Y} \end{array}\right]=G\left[\begin{array}{cc} 1 & \tan2\delta -\tan2\lambda \\ \tan2\delta +\tan2\lambda & 1+\Delta G \end{array}\right]\left[\begin{array}{c} u_{amp\_X}\\ u_{amp\_Y} \end{array}\right]$$

Then, the non-ideal amplitude control forces can be decomposed into the decayed amplitude control force $F_{amp\_a}$, and the additional virtual precession control force $F_{vir\_a}$, which can be expressed as follows:

(24) $$\left[\begin{array}{c} F_{amp\_a}\\ F_{vir\_a} \end{array}\right]=\left[\begin{array}{cc} \cos2\theta & \sin2\theta \\ -\sin2\theta & \cos2\theta \end{array}\right]\left[\begin{array}{c} F_{amp\_X}\\ F_{amp\_Y} \end{array}\right]$$

This can be obtained with further re-organization:

(25) $$\left\{\begin{array}{l} F_{amp\_a}=-Gu_{amp}u_{s}\left(1+\frac{1}{2}\Delta G-\frac{1}{2}\Delta G\cos4\theta +\tan2\delta \sin4\theta \right)\\ F_{vir\_a}=-Gu_{amp}u_{s}\left(\tan2\lambda +\frac{1}{2}\Delta G\sin4\theta +\tan2\delta \cos4\theta \right) \end{array}\right.$$

Similarly, as shown in Equation (4), without considering the unbalanced error in the driving chain, the virtual precession control signal obtained by vector reallocation of the RI-HRG can be expressed as follows:

(26) $$\left\{\begin{array}{l} u_{vir\_X}=u_{vir}u_{s}\sin2\theta \\ u_{vir\_Y}=-u_{vir}u_{s}\cos2\theta \end{array}\right.$$

The control forces applied to the driving electrodes in Channel X and Channel Y that are generated by the virtual precession control signals with the impact of the unbalanced error can be expressed as follows:

(27) $$\left[\begin{array}{c} F_{vir\_X}\\ F_{vir\_Y} \end{array}\right]=G\left[\begin{array}{cc} 1 & \tan2\delta -\tan2\lambda \\ \tan2\delta +\tan2\lambda & 1+\Delta G \end{array}\right]\left[\begin{array}{c} u_{vir\_X}\\ u_{vir\_Y} \end{array}\right]$$

With the consideration of the unbalanced error, the additional amplitude control force $F_{amp\_v}$ and the decayed virtual precession control force $F_{vir\_v}$, which are generated by the virtual precession control signals, are as follows:

(28) $$\left[\begin{array}{c} F_{amp\_v}\\ F_{vir\_v} \end{array}\right]=\left[\begin{array}{cc} \cos2\theta & \sin2\theta \\ -\sin2\theta & \cos2\theta \end{array}\right]\left[\begin{array}{c} F_{vir\_X}\\ F_{vir\_Y} \end{array}\right]$$

Further derivation is as follows:

(29) $$\left\{\begin{array}{l} F_{amp\_v}=-Gu_{vir}u_{s}\left(\frac{1}{2}\Delta G\sin4\theta -\tan2\lambda +\tan2\delta \cos4\theta \right)\\ F_{vir\_v}=-Gu_{vir}u_{s}\left(1+\frac{1}{2}\Delta G+\frac{1}{2}\Delta G\cos4\theta -\tan2\delta \sin4\theta \right) \end{array}\right.$$

By combining Equations (25) and (29), the actual amplitude control force and virtual precession control force with the impact of an unbalanced error in the driving chain can be obtained as follows:

(30) $$\left\{\begin{array}{l} F_{amp}=-Gu_{s}\left(k_{a\_a}u_{amp}+k_{v\_a}u_{vir}\right)\\ F_{vir}=-Gu_{s}\left(k_{a\_v}u_{amp}+k_{v\_v}u_{vir}\right)\\ k_{a\_a}=1+\frac{1}{2}\Delta G-\frac{1}{2}\Delta G\cos4\theta +\tan2\delta \sin4\theta \\ k_{v\_a}=\frac{1}{2}\Delta G\sin4\theta -\tan2\lambda +\tan2\delta \cos4\theta \\ k_{a\_v}=\tan2\lambda +\frac{1}{2}\Delta G\sin4\theta +\tan2\delta \cos4\theta \\ k_{v\_v}=1+\frac{1}{2}\Delta G+\frac{1}{2}\Delta G\cos4\theta -\tan2\delta \sin4\theta \end{array}\right.$$

As shown in Equation (30), due to the unbalanced error in the driving chain, the actual virtual precession control force is composed of two parts, the decayed virtual precession control force generated by the virtual precession control signal and the additional virtual precession control force generated by the amplitude control signal, which will lead to a deviation between the actual and the expected virtual precession rate. This will directly affect the output of the RI-HRG, thereby affecting the overall performance. Moreover, the angular rate output of the RI-HRG with the external angular rate Ω input can be further expressed as:

(31) $$\dot{\theta }=-k_{g}\Omega +\frac{1}{4}\Delta \left(\frac{1}{\tau }\right)\sin4\left(\theta -\theta_{\tau }\right)+\frac{1}{4}\Delta \omega \frac{Q}{E}\cos4\left(\theta -\theta_{\omega }\right)-\frac{F_{vir}}{2\omega \sqrt{E}}$$

where, $k_{g}$ is the scale factor of RI-HRG, $\Delta \left(\frac{1}{\tau }\right)$ represents the uneven damping of the resonator, $\theta_{\tau }$ is the azimuth of the damping axis, Δω represents the uneven frequency of the resonator, and $\theta_{\omega }$ is the azimuth of the frequency axis.

From Equations (30) and (31), it can be concluded that the virtual precession control force with the impact of unbalanced error in the driving chain will have a direct impact on the angular rate output of the RI-HRG. Generally, the virtual precession control signal is a constant value and the amplitude control signal is mainly influenced by the physical characteristics of the resonator and is related to the standing wave azimuth. Therefore, the identification of unbalanced errors can be achieved based on the angular rate output. In addition, the uneven damping of the resonator caused by the manufacturing process can also lead to a non-ideal angular rate output, but the effect is related to the standing wave azimuth, so that it is convenient for compensation. Nonetheless, it requires high-precision control of the RI-HRG, further reflecting the importance of the identification and compensation for the unbalanced error in the driving chain.

### 4.2. The Identification and Compensation Method of Unbalanced Error in Driving Chain

By further organizing Equations (30) and (31), the angular rate output of the RI-HRG can be divided into four parts, shown as follows:

(32) $$\left\{\begin{array}{l} \dot{\theta }=-k\Omega +\omega_{H}+\omega_{amp}+\omega_{vir}\\ \omega_{H}=\frac{1}{4}\Delta \left(\frac{1}{\tau }\right)\sin4\left(\theta -\theta_{\tau }\right)+\frac{1}{4}\Delta \omega \frac{Q}{E}\cos4\left(\theta -\theta_{\omega }\right)\\ \omega_{amp}=k_{vir}u_{amp}\left(\tan2\lambda +\frac{1}{2}\Delta G\sin4\theta +\tan2\delta \cos4\theta \right)\\ \omega_{vir}=k_{vir}u_{vir}\left(1+\frac{1}{2}\Delta G+\frac{1}{2}\Delta G\cos4\theta -\tan2\delta \sin4\theta \right) \end{array}\right.$$

where, $\omega_{H}$ is the angular rate drift caused by the physical defects of the hemispherical resonator, $\omega_{amp}$ is the angular rate drift caused by the amplitude control signal with the impact of the unbalanced error, $\omega_{vir}$ is the angular rate caused by the actual control force for virtual precession, and $k_{vir}$ is the gain between the angular rate to the virtual precession control signal.

As shown in Equation (32), the angular rate drift caused by the physical defects of the resonator is only related to the standing wave azimuth, while it is independent of the multi-loop control signals. Besides, to obtain a small angular rate output, the virtual precession control signal is much smaller than the amplitude control signal, so that the impact of the non-ideal amplitude control forces that are caused by the virtual precession control signal can be ignored. On this basis, without the external angular rate input, a couple of reversal virtual precession control forces with the same amplitude are applied to the RI-HRG, driving the standing wave to rotate at a small angular rate in opposite directions, and the angular rate outputs can be expressed as follows:

(33) $$\left\{\begin{array}{c} {\dot{\theta }}_{vir\_p}=\omega_{H}+\omega_{amp}+k_{vir}u_{vir}\left(1+\frac{1}{2}\Delta G+\frac{1}{2}\Delta G\cos4\theta -\tan2\delta \sin4\theta \right)\\ {\dot{\theta }}_{vir\_n}=\omega_{H}+\omega_{amp}-k_{vir}u_{vir}\left(1+\frac{1}{2}\Delta G+\frac{1}{2}\Delta G\cos4\theta -\tan2\delta \sin4\theta \right) \end{array}\right.$$

Denoting the difference of the angular rate output with the application of a couple of reversal virtual precession control signals as ${\dot{\theta }}_{vir\_dif}$, it can be expressed as follows:

(34) $${\dot{\theta }}_{vir\_dif}=2k_{vir}u_{vir}\left(1+\frac{1}{2}\Delta G+\frac{1}{2}\Delta G\cos4\theta -\tan2\delta \sin4\theta \right)$$

According to Equation (34), ${\dot{\theta }}_{vir\_dif}$ is independent of the physical defects and amplitude control signal, while is related to the unbalanced gain error ΔG and the equivalent misalignment angle 2δ, so that the unbalanced gain error ΔG and the equivalent misalignment angle 2δ can be identified through the least squares method with ${\dot{\theta }}_{vir\_dif}$.

Further, the sum of the angular rate output is denoted as ${\dot{\theta }}_{vir\_sum}$, shown as follows:

(35) $${\dot{\theta }}_{vir\_sum}=2\omega_{H}+2\omega_{amp}=2\omega_{H}+2k_{vir}u_{amp}\left(\tan2\lambda +\frac{1}{2}\Delta G\sin4\theta +\tan2\delta \cos4\theta \right)$$

As shown in Equation (35), only the non-ideal angular rate caused by the unbalanced equivalent misalignment angle error 2λ is a constant deviation, while the non-ideal angular rate caused by the rest factors are all periodic terms related to the standing wave azimuth. Therefore, the unbalanced equivalent misalignment angle error 2λ can be identified by extracting the ${\dot{\theta }}_{vir\_sum}$ according to the standing wave azimuth with the entire period.

Based on Equations (34) and (35), the identification and compensation method of the unbalanced error in driving chain can be obtained. The steps are as follows:

1. Applying a couple of reversal virtual precession control signals with the same amplitude to the RI-HRG to obtain a couple of small angular rate outputs. Recording the amplitude control signal $u_{amp}$, virtual precession control signal $u_{vir}$, standing wave azimuth θ, and the angular rate outputs, ${\dot{\theta }}_{vir\_p}$ and ${\dot{\theta }}_{vir\_n}$;
2. Calculating the difference of the angular rate output ${\dot{\theta }}_{vir\_dif}$, then using the least squares method to obtain the estimated values of the unbalanced gain error $\Delta \hat{G}$ and the equivalent misalignment angle $2\hat{\delta }$ based on Equation (34) and further recording the estimated value of the gain between the angular rate to the virtual precession control signal ${\hat{k}}_{vir}$;
3. Calculating the sum of the angular rate output ${\dot{\theta }}_{vir\_sum}$, extracting the data according to the standing wave azimuth of the resonator with the entire period, then calculating the estimated value of the unbalanced equivalent misalignment angle error $2\hat{\lambda }$ based on Equation (35) and the estimated value of the gain between the angular rate to the virtual precession control signal ${\hat{k}}_{vir}$;
4. With the identification results of the unbalanced error in the driving chain, an error compensation matrix can be derived based on Equation (21) and applied in the digital controller to realize the compensation of the unbalanced error in the driving chain, the error compensation matrix is as follows:
  (36) $$\left[\begin{array}{c} {\tilde{u}}_{Ctrl\_X\_O}\\ {\tilde{u}}_{Ctrl\_Y\_O} \end{array}\right]=\left[\begin{array}{cc} \frac{1+\Delta \hat{G}}{1+\Delta \hat{G}-\tan^{2}2\hat{\delta }+\tan^{2}2\hat{\lambda }} & \frac{\tan2\hat{\lambda }-\tan2\hat{\delta }}{1+\Delta \hat{G}-\tan^{2}2\hat{\delta }+\tan^{2}2\hat{\lambda }}\\ -\frac{\tan2\hat{\delta }+\tan2\hat{\lambda }}{1+\Delta \hat{G}-\tan^{2}2\hat{\delta }+\tan^{2}2\hat{\lambda }} & \frac{1}{1+\Delta \hat{G}-\tan^{2}2\hat{\delta }+\tan^{2}2\hat{\lambda }} \end{array}\right]\left[\begin{array}{c} u_{Ctrl\_X\_O}\\ u_{Ctrl\_Y\_O} \end{array}\right]$$

The above completes the identification of and compensation for the unbalanced error in the driving chain for the RI-HRG. It can be seen from the error compensation matrix that the compensation effect can be further improved by iteratively using the identification and compensation method as long as the circuit resolution and time cost permit.

## 5. The Simulation and Experiments

The multi-loop control system used in the RI-HRG that realizes the stable control of the vibration mode of the hemispherical resonator plays an important role in the performance of the RI-HRG. Furthermore, the virtual precession control in the control system of RI-HRG is also an important component, which can be further used to suppress and compensate for the impact of the physical defects of the resonator. The identification and compensation method of the unbalanced error in the driving chain can effectively improve the control accuracy of the multi-loop control of the RI-HRG, which is verified with the simulation and experiments in kind, providing a basis for subsequent error compensation of the gyroscope. Furthermore, with the help of digital control circuits and software, the identification and compensation of the unbalanced error in the driving chain can be automatically completed, further improving the accuracy and efficiency of the error identification and compensation.

### 5.1. The Simulation

**Figure 11:**
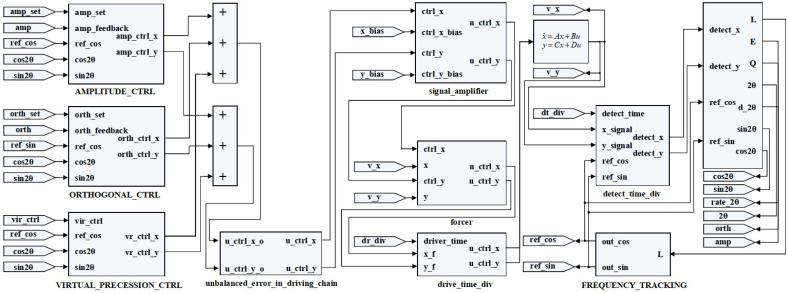
The numerical simulation platform for unbalanced error in driving chain for RI-HRG.

A numerical simulation platform was built with consideration of the multi-loop control scheme and key characteristics of the RI-HRG to better verify the correctness of the model of the unbalanced error in the driving chain and the effectiveness of the error identification and compensation method by comparing the error identification results with the preset errors through the numerical simulation. The numerical simulation platform is shown in Figure 11.

Besides, the key characteristics and the preset errors used in the simulation are shown in Table 1. The key characteristics used in the simulation such as the inner spherical radius and the outer spherical radius are obtained from the document of the structure design. In addition, the preset errors were obtained from the previous experimental results and the estimated results of the unbalanced error in the detection chain, the main factors of which are similar to the unbalanced error in the driving chain.

**Figure 12:**
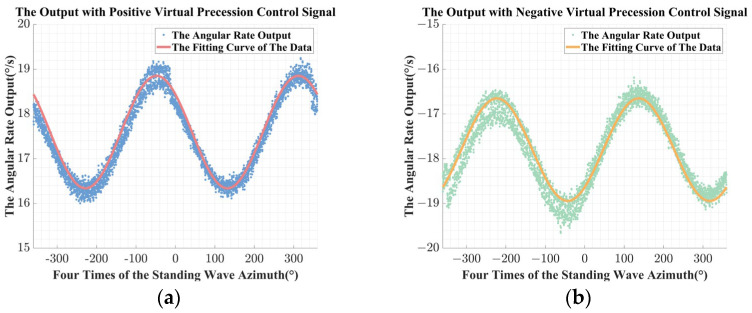

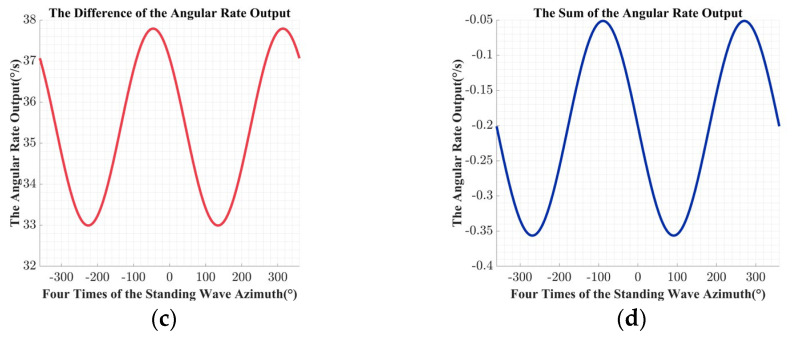
The simulation results with the unbalanced error in driving chain: (**a**) the angular rate output with the positive virtual precession control signal; (**b**) the angular rate output with the negative virtual precession control signal; (**c**) the difference of the angular rate output; (**d**) the sum of the angular rate output.

As shown in Equation (32), the impact of the unbalanced error in the driving chain on the angular rate output is related to the four times standing wave azimuth. Therefore, in subsequent simulations and experiments, the main focus is on the relationship between the angular rate output and the four times standing wave azimuth. On this basis, with the consideration of the unbalanced error in the driving chain, the simulation results obtained with a couple of virtual precession control signals that are opposite to each other were applied are shown in Figure 12.

**Figure 13:**
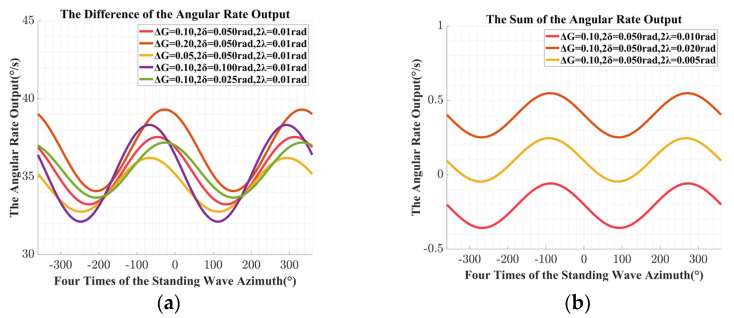
The simulation results with different unbalanced error in driving chain: (**a**) the difference of the angular rate output with different unbalanced gain error and the equivalent misalignment angle; (**b**) the sum of the angular rate output with different unbalanced equivalent misalignment angle error.

In the actual data processing, due to the discrete sampling process, it is difficult to directly achieve the sum and difference operation of multiple sets of the angular rate outputs. Therefore, based on Equations (32) and (33), the obtained discrete data points are first fitted, and then the corresponding operation required for identification is completed with the fitting results, as is shown in Figure 12. Besides, according to Figure 12, the following results could be obtained:

1. The estimated value of the unbalanced gain error $\Delta \hat{G}$ obtained from the simulation is 0.0996, and the relative deviation from the preset value is −0.3970%;
2. The estimated value of the equivalent misalignment angle $2\hat{\delta }$ obtained from the simulation is 0.0508 rad, which has a relative deviation of 1.6008% from the preset value;
3. The estimated value of the unbalanced equivalent misalignment angle error $2\hat{\lambda }$ obtained from the simulation is 0.0116 rad, while the relative deviation from the preset value is 12.5175%;

From the simulation results, the accuracy of the model of the unbalanced error in driving chain is verified, however, the accuracy of the estimated value of the unbalanced equivalent misalignment angle error obtained in the simulation is slightly insufficient, which is related to the control signal of the amplitude control loop in the simulation platform. The closed-loop control in the simulation platform is difficult to fully replicate in the actual systems; although the amplitude can be achieved, the accuracy is not enough for further analysis. Besides, in order to better verify the impact of the unbalanced error on the angular rate output as conducted in Equation (32), the preset error parameters in the simulation were further adjusted, and the simulation results were compared. The difference of the angular rate output can better reflect the impact of the variation on the unbalanced gain error ΔG and the equivalent misalignment angle 2δ on the angular rate output, while the sum of the angular rate output can better reflect the non-ideal angular rate output caused by the unbalanced equivalent misalignment angle error 2λ. Therefore, further simulation results are presented as follows:

As shown in Figure 13a, the amplitude of the difference of the angular rate output obtained with a couple of virtual precession control signals that are reverse to each other increases with the unbalanced gain error ΔG and the equivalent misalignment angle 2δ. Besides, the phase relative to the four times of the standing wave azimuth is also changed. The simulation results in Figure 13b show that the average of the sum of the angular rate output also increases with the unbalanced equivalent misalignment angle error 2λ, and there is also a small to negligible change in the phase relative to the standing wave azimuth, which is the result of the amplitude control variable being affected by the non-ideal physical characteristics of the hemispherical resonator in the simulation model.

The phenomenon in the simulation results shown in Figure 13 is basically consistent with the description in Equations (31) and (32). Therefore, further experiments in kind were conducted to further verify the effectiveness of the identification and compensation method.

### 5.2. Experiments in Kind

**Figure 14:**
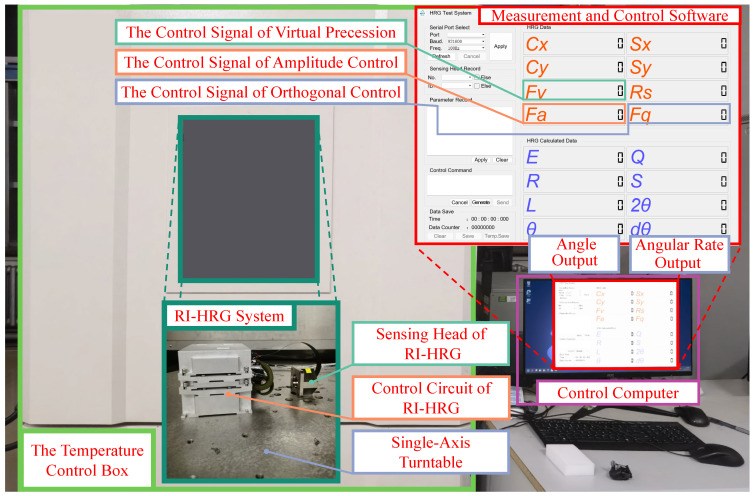
The experimental system and the measurement and control software.

The RI-HRG system used in the experiment mainly consists of two parts, namely the sensing head and the control circuit. According to the previous performance testing of the sensing head, the quality factor of the sensing head used in the experiment was about 7.91 × 10^6^, and the frequency split was about 3.1 MHz During the experiment, the external angular rate input was not required; however, a single-axis turntable was used to keep the sensitive axis of the sensing head to the east, so that the influence of the Earth rotation rate was eliminated, and a temperature control box was used to isolate the influence of the temperature. Besides, according to the experimental process, measurement and control software was used to modify the virtual precession control signal and record the relevant data. Furthermore, a temperature control box was also used to isolate the impact of the changes in the external environment. The experimental system and the measurement and control software are shown in Figure 14.

The identification and compensation method of the unbalanced error in the driving chain for RI-HRG are as follows:

1. Install the sensing head and the control circuit of RI-HRG on the surface of the single-axis turntable, ensuring the sensitive axis of the sensing head is perpendicular to the rotation axis of the turntable and the stability of the installation, turn on the temperature control box to ensure the stability of the experimental environment;
2. Rotate the single-axis turntable to keep the sensitive axis of the sensing head pointing to the east;
3. Open the measurement and control software, turn on the DC power, and connect the control circuit of the RI-HRG through serial port; on this basis, observe the data of the RI-HRG transmitted by the control circuit, as shown in the software, to ensure the full preheating of the RI-HRG;
4. After the preheating of the RI-HRG, control commands are sent through the measurement and control software to apply three virtual precession control signals with the same sign but different amplitudes. Correspondingly, three sets of the virtual precession control signals, standing wave azimuth and angular rate output are recorded through the measurement and control software;
5. The three sets of data obtained are further extracted according to the standing wave azimuth of the resonator with the entire periods, and the average angular rate output is calculated. On this basis, estimate the proportional factor between the virtual precession control signal and the angular rate of the virtual precession through simple fitting;
6. Calculate the virtual precession control signal corresponding to the angular rate of 0.1°/s before error compensation. Send commands through the measurement and control software to sequentially apply a couple of virtual precession control signals that are opposite to each other. Record two sets of the gyro amplitude control signals, virtual precession control signals, standing wave azimuth and angular rate output through the measurement and control software;
7. Calculate the difference of the output, and the least squares method is used to obtain the estimated value of the unbalanced gain error, the equivalent misalignment angle and the gain between the angular rate to the virtual precession control signal;
8. Calculate the sum of the angular rate output and extract the data according to the standing wave azimuth with the entire period, then calculate the estimated value of the unbalanced equivalent misalignment angle error;
9. Substitute the identification results of the unbalanced error in the driving chain into Formula (35), and send the error compensation matrix to the control circuit through the measurement and control software to achieve the error compensation;
10. Repeat steps (4)–(8) to verify the compensation results of the unbalanced error in the driving chain, thereby completing the compensation for the error.

**Figure 15:**
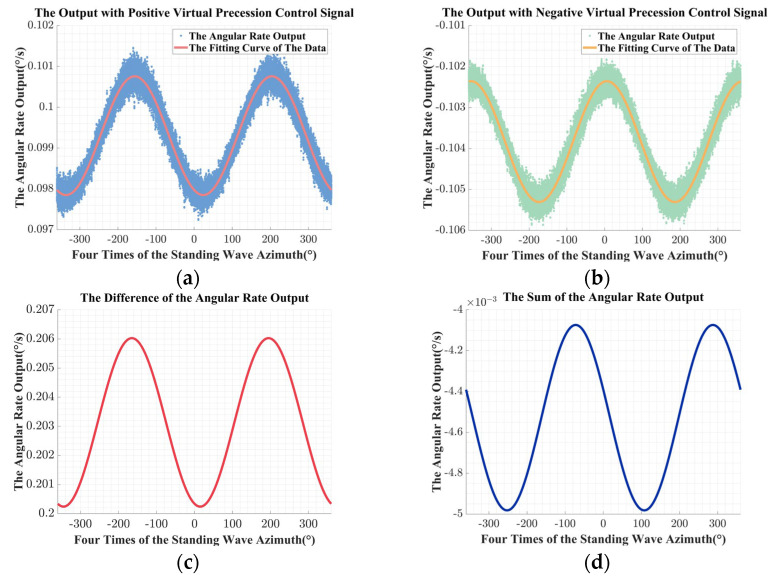
The experimental results before compensation: (**a**) the angular rate output with the positive virtual precession control signal; (**b**) the angular rate output with the negative virtual precession control signal; (**c**) the difference of the angular rate output; (**d**) the sum of the angular rate output.

According to the experimental process, the experiments in kind were conducted, and the angular rate output of the RI-HRG and the corresponding calculation results obtained before the compensation for the unbalanced error in the driving chain are shown in Figure 15.

With the angular rate output and the correspondingly calculation results, the unbalanced error in the driving chain is identified. The estimated value of the unbalanced gain error is −2.7148 × 10^−2^, the estimated value of the equivalent misalignment angle is 3.6215 × 10^−3^, and the estimated value of the unbalanced equivalent misalignment angle error is −2.1985 × 10^−2^.

**Figure 16:**
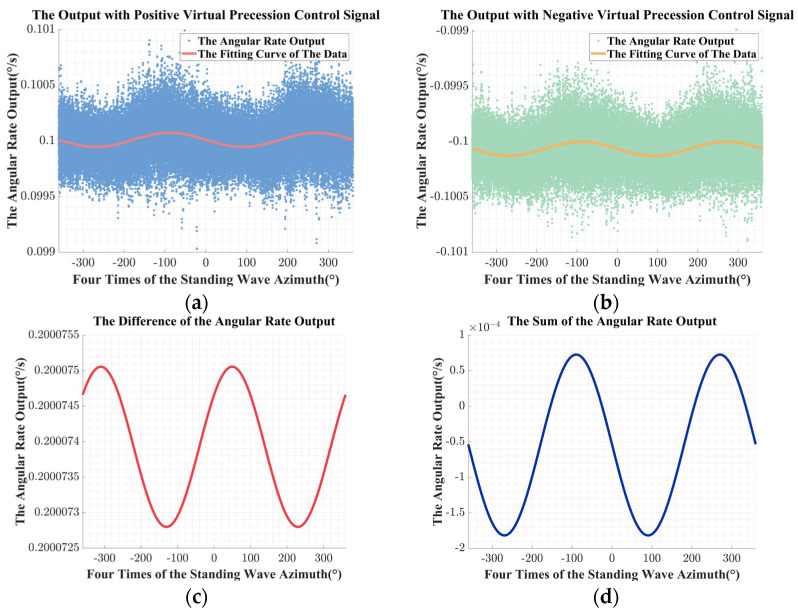
The experimental results after compensation: (**a**) the angular rate output with the positive virtual precession control signal; (**b**) the angular rate output with the negative virtual precession control signal; (**c**) the difference of the angular rate output; (**d**) the sum of the angular rate output.

Then, the error compensation matrix is calculated based on Equation (36) and applied in the digital control circuit. The experimental results after compensation are shown in Figure 16.

With the experiment results after compensation for the unbalanced error in the driving chain, the residual errors were further identified after the compensation to verify the efficiency of the identification and the compensation method. After the compensation, the estimated value of the unbalanced gain error was 7.3261 × 10^−6^, which has been compressed by over three orders of magnitude. The estimated value of the equivalent misalignment angle was −4.2887 × 10^−6^, which is nearly three orders smaller. The estimated value of the unbalanced equivalent misalignment angle error was −2.7275 × 10^−4^, which has been compressed by nearly two orders of magnitude.

Besides, comprehensively comparing Figure 15a,b and Figure 16a,b, after the compensation, the fluctuation of the angular rate output caused by the unbalanced error in the driving chain was effectively suppressed, and the peak-to-peak value of the fitting curve was compressed for about two orders of magnitude.

With the improvement in the circuit resolution, the compensation effect could be further improved by iteratively using the identification and compensation method.

**Figure 17:**
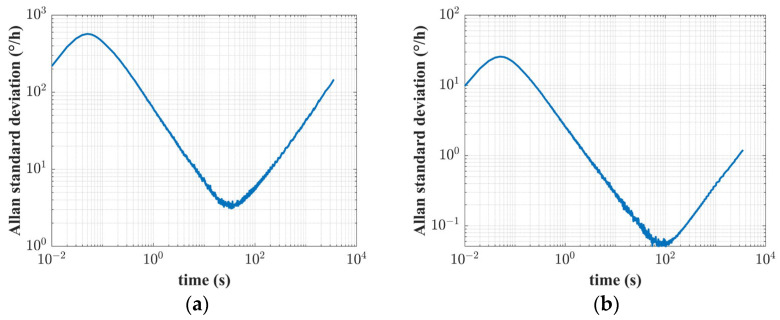
The zero-bias instability of RI-HRG: (**a**) the results before compensation; (**b**) the results after compensation.

Furthermore, as zero-bias instability is an essential indicator of the performance of RI-HRG, so that the zero-bias instability test is conducted before and after the compensation of the unbalanced error in the driving chain, and the lowest point of the Allan standard deviation curve can represent the zero-bias instability of the RI-HRG, the Allan standard deviation curves are shown in Figure 17.

As shown in Figure 17, after the compensation for the unbalanced error in the driving chain, the zero-bias instability of the RI-HRG is improved from 3.0950°/h to 0.0511°/h and the promotion is more than an order of magnitude, which further verifies the compensation effect for the unbalanced error. Furthermore, the physical characteristic defects such as damping anisotropy are angle dependent errors, and with an improvement in the control accuracy, the precision of the following compensation to the angle dependent errors will also be improved, so that the overall performance of the RI-HRG will be further enhanced.

## 6. Conclusions

In this paper, a model describing the impact of the unbalanced error in the driving chain on the control forces is established with the comprehensive consideration of the impact caused by the defects that are generated from the manufacturing process and the control scheme, and the error model is validated through simulation. With this foundation, the impact on the angular rate output of the RI-HRG caused by non-ideal control forces was analyzed with an emphasize on non-ideal virtual precession control forces. Thus, a couple of virtual precession control signals with reversal to each other were used to achieve the error identification, which also realized the decoupling of the impact of the angle dependent error caused by non-ideal physical characteristics of the hemispherical resonator. Further, the error compensation matrix obtained from the error identification was applied in the digital control circuit to realize the error compensation. With the help of digital control circuits and software, the process can be automatically completed, further improving the accuracy and efficiency of error identification and compensation.

A simulation and experiments were conducted to verify the effectiveness of the identification and compensation method. After the error compensation, the unbalanced gain error was suppressed from 2.7148 × 10^−2^ to 7.3261 × 10^−6^, the equivalent misalignment angle was suppressed from 3.6215 × 10^−3^ to −4.2887 × 10^−6^, the unbalanced equivalent misalignment angle error was suppressed from −2.1985 × 10^−2^ to −2.7275 × 10^−4^, and the key performance indicator of the RI-HRG, the zero-bias instability, was improved from 3.0950°/h to 0.0511°/h. These experiment results have confirmed the effectiveness of the proposed method. Besides, as the impact of the unbalanced error is significantly suppressed, the precision of the compensation to the angle dependent errors will also be improved in the future, thereby further improving the overall performance of the RI-HRG. Meanwhile, this method is also applicable to CVGs operating in rate-integrating mode.

## Figures and Tables

**Table 1:** The key characteristics and the preset errors used in the simulation.

Symbol	Definition	Value
$R_{1}$	The inner spherical radius	9.65 × 10^−3^ m
$R_{2}$	The outer spherical radius	10.35 × 10^−3^ m
d	The distance between the lip-edge and the electrode	15 × 10^−6^ m
$u_{DC}$	The DC high voltage	200 V
$u_{vir}$	The virtual precession control signal	1 V
ΔG	The unbalanced gain error	0.1
2δ	The equivalent misalignment angle	0.05 rad
2λ	The unbalanced equivalent misalignment angle error	0.01 rad

## Data Availability

All data are contained within the article.
